# Hydrogen Sulfide Improves Endothelial Dysfunction via Downregulating BMP4/COX-2 Pathway in Rats with Hypertension

**DOI:** 10.1155/2016/8128957

**Published:** 2016-08-23

**Authors:** Lin Xiao, Jing-Hui Dong, Sheng Jin, Hong-Mei Xue, Qi Guo, Xu Teng, Yu-Ming Wu

**Affiliations:** ^1^Department of Physiology, Hebei Medical University, Shijiazhuang 050017, China; ^2^Hebei Key Laboratory of Animal Science, Hebei Medical University, Shijiazhuang 050017, China; ^3^Hebei Collaborative Innovation Center for Cardio-Cerebrovascular Disease, Shijiazhuang 050017, China; ^4^Key Laboratory of Vascular Medicine of Hebei Province, Shijiazhuang 050017, China

## Abstract

*Aims*. We object to elucidate that protective effect of H_2_S on endothelium is mediated by downregulating BMP4 (bone morphogenetic protein 4)/cyclooxygenase- (COX-) 2 pathway in rats with hypertension.* Methods and Results*. The hypertensive rat model induced by two-kidney one-clip (2K1C) model was used. Exogenous NaHS administration (56 *μ*mol/kg/day, intraperitoneally once a day) reduced mean arterial pressure (MAP) of 2K1C rats from 199.9 ± 3.312 mmHg to 159.4 ± 5.434 mmHg, while NaHS did not affect the blood pressure in the Sham rats and ameliorated endothelium-dependent contractions (EDCs) of renal artery in 2K1C rats. 2K1C reduced CSE level twofold, decreased plasma levels of H_2_S about 6-fold, increased BMP4, Nox2, and Nox4 levels 2-fold and increased markers of oxidative stress MDA and nitrotyrosine 1.5-fold, upregulated the expression of phosphorylation-p38 MAPK 2-fold, and increased protein levels of COX-2 1.5-fold, which were abolished by NaHS treatment.* Conclusions*. Our results demonstrate that H_2_S prevents activation of BMP4/COX-2 pathway in hypertension, which may be involved in the ameliorative effect of H_2_S on endothelial impairment. These results throw light on endothelial protective effect of H_2_S and provide new target for prevention and therapy of hypertension.

## 1. Introduction

Hydrogen sulfide (H_2_S) has been proved to be the third endogenous gasotransmitter following nitric oxide (NO) and carbon oxide (CO). H_2_S is endogenously produced from L-cysteine by two pyridoxal-5′-phosphate-dependent enzymes, that is, cystathionine *β*-synthase (CBS) and cystathionine *γ*-lyase (CSE) in mammalian tissues [1]. Recently, it was found that another mitochondrial enzyme, 3-mercaptopyruvate sulfurtransferase (3-MST) in conjunction with cysteine aminotransferase (CAT), contributes significantly in generating H_2_S from L-cysteine in the presence of *α*-ketoglutarate [2]. The expression of those three enzymes is tissue-specific, and, in blood vessels, CSE is a major H_2_S-producing enzyme expressed in both smooth muscle and endothelium [3–5]. H_2_S is endowed with biological and physiological functions in cardiovascular system. H_2_S has been well known as a vasodilator and plays an integral role in the homeostatic regulation of blood pressure [6].

Endothelial dysfunction is an initial factor in the pathogenesis of various vascular diseases such as atherosclerosis and hypertension. A number of studies have showed that H_2_S played endothelium protection through decreasing the level of oxidative stress [7–10], strengthening endothelial NO production via activating eNOS Ser 1177 phosphorylation [11], and inhibiting inflammation of endothelium, which resulted in ameliorating the development of hypertension [12]. However, the exact mechanism of H_2_S remains to be fully clarified.

Bone morphogenetic protein 4 (BMP4) is one of the BMP family from BMP2 to BMP7, which belongs to TGF-*β* superfamily [13]. Original studies showed that BMP4 was bound to BMP receptors containing type I and type II [14] and then regulated physiological and pathological process of embryonic development, bone, and cartilage formation [15–17]. Several studies have further implied that BMP4 might be involved in exaggerating cardiac ischemia-reperfusion injury [18] and atherosclerotic calcification plaques [19]. Activating vascular BMP4 can promote vascular calcification in hyperglycemia and diabetes [20]. BMP4 infused chronically results in hypertension and is considered as a novel mediator of endothelial dysfunction and hypertension [21]. Endothelial dysfunction can be ameliorated via inhibiting BMP4 cascade [22].

As a proinflammatory gene, BMP4 induces endothelium dysfunction in systemic circulation and resulted in not only impairment of vascular relaxation [23] but also exaggeration of vascular contraction. BMP4 binds to BMP4 receptor which activates NADPH oxidase and then chronically increased the expression of cyclooxygenase- (COX-) 2 through p38 MAPK-dependent mechanism. COX-2 contributes to production of constrictive prostaglandins followed by impairing endothelial function and exacerbating endothelium-dependent contractions (EDCs) [24].

Our previous results found that H_2_S lowered blood pressure and improved endothelial function [25]. However, the detailed mechanism of ameliorative effect of H_2_S on impaired endothelial function in hypertension still remained underlying. Therefore, we hypothesize that H_2_S can downregulate BMP4/COX-2 pathway, which may be involved in ameliorating endothelial dysfunction in hypertension. Here, the rat model of hypertension resulting from two-kidney one-clip (2K1C) model is used to verify the effect of H_2_S on BMP4/COX-2 pathway and EDCs in renal artery.

## 2. Methods

### 2.1. Agents

NaHS, ACh, and* L*-NAME were purchased from Sigma-Aldrich Chemical (St. Louis, MO, United States). ACh and* L*-NAME were dissolved in distilled water.

### 2.2. Preparation of Hypertensive Model in Rats

7-week-old male Sprague-Dawley rats were obtained from Animal Research Center of Hebei Medical University, which were kept in ordinary cages at room temperature of 25 ± 3°C with 12 h dark/light cycles (lights on 6:00) with food and water ad libitum. All animal procedures were complied with the Animal Management Rule of the Ministry of Health, People's Republic of China (documentation number 55, 2001), and the Care and Use of Laboratory Animals published by the US National Institutes of Health (NIH Publication number 85-23, revised in 1996) and approved by the Animal Care Committee of Hebei Medical University.

The rats were randomly divided into 4 groups (*n* = 6): Sham, Sham + NaHS, 2K1C, and 2K1C + NaHS. The rats in 2K1C and 2K1C + NaHS group were anesthetized with intraperitoneal injections of pentobarbital sodium (30 mg/kg) and then were subjected to unilateral clipping of the renal artery to establish 2K1C model. In brief, a left kidney was exposed via laparotomy and then the left renal artery was carefully separated from the left renal vein and connected tissues. In the 2K1C and 2K1C + NaHS groups, the left renal artery was clipped by a rigid U-shaped solid silver clip with an open slit of 0.25 mm, resulting in partial occlusion of renal perfusion. The contralateral kidney was left untouched. Sham and Sham + NaHS groups underwent the same procedure, but kidneys were only mobilized and renal vessels were only separated instead of being partially ligated. The rats were kept in cages after surgery for three weeks until blood pressure was stable; Sham + NaHS and 2K1C + NaHS groups received NaHS 56 *μ*mol/kg/day intraperitoneally from the fourth week after the surgery which maintained for 20 weeks. Sham and 2K1C groups received saline as vehicle.

### 2.3. Mean Arterial Pressure Measurement

Mean arterial pressure (MAP) was measured noninvasively by tail-cuff plethysmography (BP-100A, Chengdu Taimeng Software CO. Ltd., Chengdu, China) after the rats were stabilized and remained quiescent. Briefly, MAP was measured before and further at every four weeks after surgery for 20 weeks. MAP measurement was always conducted between 9:00 and 12:00 AM and an average of 3 consecutive readings was taken as the systolic blood pressure of each rat.

### 2.4. Blood Vessel Preparation

Adult male rats were euthanized by CO_2_ suffocation and rat intralobar renal arteries were dissected and placed in ice-cold Krebs solution (mmol/L): 119 NaCl, 4.7 KCl, 2.5 CaCl_2_, 1 MgCl_2_, 25 NaHCO_3_, 1.2 KH_2_PO_4_, and 11 D-glucose, which gassed by 95% O_2_/5% CO_2_ at 37°C (pH~7.4). The arteries were carefully cleaned of adhering adipose tissue and cut into ring segments 2 mm in length for functional studies. Rings were suspended in a myograph (620 M, Danish MyoTechnology, Aarhus, Denmark) for recording of changes in isometric tension. Briefly, 2 stainless steel wires (40 *μ*m in diameter) were put through the lumen of the vessel, and each wire was fixed to the jaws built in the myograph. The organ chamber was filled with 5 mL of Krebs solution. Each ring was stretched to an optimal tension of 2.5 mN and then allowed to stabilize for 60 minutes before the start of each experiment.

### 2.5. Endothelial Functional Studies

The series of experiments examined the alterations in EDCs. Firstly, renal artery rings were treated for 30 minutes with 100 *μ*mol/L L-NAME to eliminate the interference of endothelium-derived nitric oxide (NO), a procedure commonly adopted to uncover ACh-induced EDCs [26, 27], and then contractions were elicited by Ach (0.03–100 *μ*mol/L). Vasocontraction was determined in relative values as the percentage of 60 mmol/L KCl contraction.

### 2.6. Measurement of H_2_S Content

H_2_S levels in plasma were measured as described in previous experiment [28]. Briefly, 30 *μ*L plasma was used to detect H_2_S. H_2_S concentrations were determined using a curve generated with sodium sulfide (0–40 *μ*mol/L) standards, and the H_2_S concentration in plasma was expressed as *μ*mol/L.

### 2.7. Western Blot Analysis

Renal arteries from four groups were homogenized in ice-cold RIPA lysis buffer (1 *μ*g/mL leupeptin, 5 *μ*g/mL aprotinin, 100 *μ*g/mL PMSF, 1 mmol/L sodium orthovanadate, 1 mmol/L EGTA, 1 mmol/L EDTA, 1 mmol/L NaF, and 2 mg/mL *β*-glycerophosphate). The homogenates were incubated on ice for 20 minutes and then centrifuged at 20 000 ×g for 20 minutes at 4°C. The supernatant was collected and the protein concentration was determined using the bicinchoninic acid (BCA) method (Generay biotechnology, Shanghai, China). Equal amounts of protein samples were electrophoresed through a 7.5% SDS-polyacrylamide gel and then transferred onto immobilon-P polyvinylidene difluoride (PVDF) membrane (Millipore) using wet transfer at 100 V for 90 minutes at 4°C. Nonspecific binding sites were blocked by 5% nonfat milk or 1% BSA in 0.05% Tween-20 Tris-buffered saline (TBST) and then incubated overnight at 4°C with primary antibodies, anti-AT1R (1 : 1000, Abcam), anti-BMP4 (1 : 500, Sigma), anti-Nox2 (1 : 1000, Abcam), anti-Nox4 (1 : 1000, proteintech), anti-p67^phox^ (1 : 1000, EPITOMICS), anti-Nitrotyrosine (1 : 1000, MILLIPORE), COX-2 (1 : 1000, Cayman), CSE (1 : 1000, Proteintech), and p38 MAPK, phospho-p38 MAPK (1 : 1000, Wanleibio). The blots were incubated with appropriate secondary antibodies with a horseradish peroxidase- (HRP-) conjugated goat anti-rabbit antibody (Proteintech, Chicago, United States) or HRP-conjugated rabbit anti-goat antibody (Proteintech, Chicago, United States) at 1 : 3000 dilution for 1 hour at room temperature. All blots washes were performed in TBST. Blots were developed with an enhanced chemiluminescence detection system (Sagecreation, Beijing, China). Densitometry was performed using lane-1 system (Sagecreation, Beijing, China).

### 2.8. Measurement of Malondialdehyde (MDA) Concentration

MDA concentration in plasma was measured by using thiobarbituric acid reactive substances (TBARS) assay kit (Nanjing Jiancheng Bioengineering Institute, Nanjing, Jiangsu, China) according to the instruction of manufacturer. The plasma was mixed with working solution, followed by 40-minute incubation in a boiling water bath. The mixed solution was centrifuged at 3500 rpm for 10 minutes. The absorbance of the supernatant (532 nm) was measured. The results were expressed as nmol/mL.

### 2.9. Statistical Analysis

Data are represented as means ± SEM. *E*_max_ denotes the maximal response produced by the constrictor or dilator. Statistical significance was determined by one-way analysis of variance (ANOVA) followed by Bonferroni post hoc tests (GraphPad Software, San Diego, United States). *P* values less than 0.05 indicate statistical significance.

## 3. Result

### 3.1. Exogenous Administration of NaHS Lowered Blood Pressure and Ameliorated EDCs in Renal Artery in Hypertensive Rats

**Figure 1:**
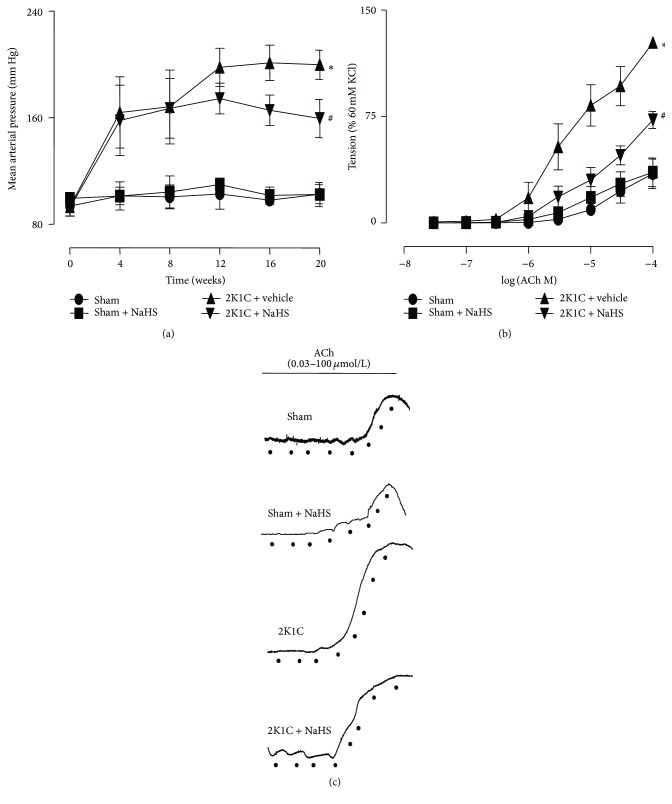
Effect of NaHS on blood pressure and endothelial-dependent renal arterial contraction. (a) Mean arterial pressure (MAP); (b) endothelium-dependent contractions (EDCs) in four groups; (c) original recording of EDCs in four groups. Data are means ± SEM. ^*∗*^ *P* < 0.05 versus Sham; ^#^ *P* < 0.05 versus 2K1C vehicle. *n* = 10 in each group for MAP measurement; *n* = 6 in each group for EDCs measurement.

In 2K1C rats, NaHS (56 *μ*mol/kg/day) treatment for 20 weeks significantly lowered MAP (159.4 ± 5.434 mmHg versus 199.9 ± 3.312 mmHg, *P* < 0.05). MAP in Sham + NaHS rats had no changes compared with Sham rats (102.6 ± 2.687 versus 102.4 ± 3.721 mmHg) (Figure 1(a)). Rings in 2K1C rats displayed enhancing EDCs compared with Sham rings (*E*_max_: 127.2 + 3.216% in 2K1C rings, *n* = 7 versus 34.68 + 10.34% in Sham, *n* = 5; *P* < 0.05). Renal arteries from 2K1C + NaHS rats reduced the enhanced EDCs to 72.66 + 6.007%. Chronically exogenously administrating NaHS had no effect on EDCs in Sham rats (Figure 1(b)).

### 3.2. Exogenous Administration of NaHS Improved the Level of Plasma H_2_S and Renal Artery CSE Protein Expression in Hypertensive Rats

**Figure 2:**
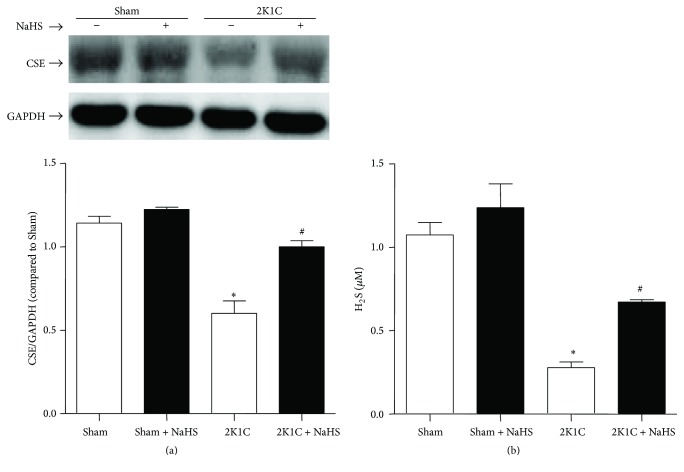
Effect of NaHS on protein levels of CSE in renal artery and plasma levels of H_2_S. (a) Protein expression of CSE in renal arteries; (b) H_2_S levels in plasma. Data are means ± SEM. ^*∗*^ *P* < 0.05 versus Sham; ^#^ *P* < 0.05 versus 2K1C vehicle. *n* = 4 in each group for Western blotting; *n* = 10 in each group for plasma H_2_S level.

Western blot analysis showed that the protein level of CSE reduced in 2K1C rats compared with Sham rats. NaHS treatment in 2K1C rats increased the expression of CSE. There is no difference among the Sham, Sham + NaHS, and 2K1C + NaHS groups (Figure 2(a)). The plasma H_2_S level in 2K1C rats was significantly lower than that in Sham rats (0.28 ± 0.04 versus 1.07 ± 0.08 *μ*mol/L, *P* < 0.05). NaHS treatment elevated the plasma H_2_S level to 0.67 ± 0.01 *μ*mol/L (*P* < 0.05). There is no difference between Sham and Sham + NaHS group (1.07 ± 0.08 versus 1.24 ± 0.14 *μ*mol/L, *P* > 0.05) (Figure 2(b)).

### 3.3. Exogenous Administration of NaHS Downregulated the Protein Expression of BMP4 in Hypertensive Rats

**Figure 3:**
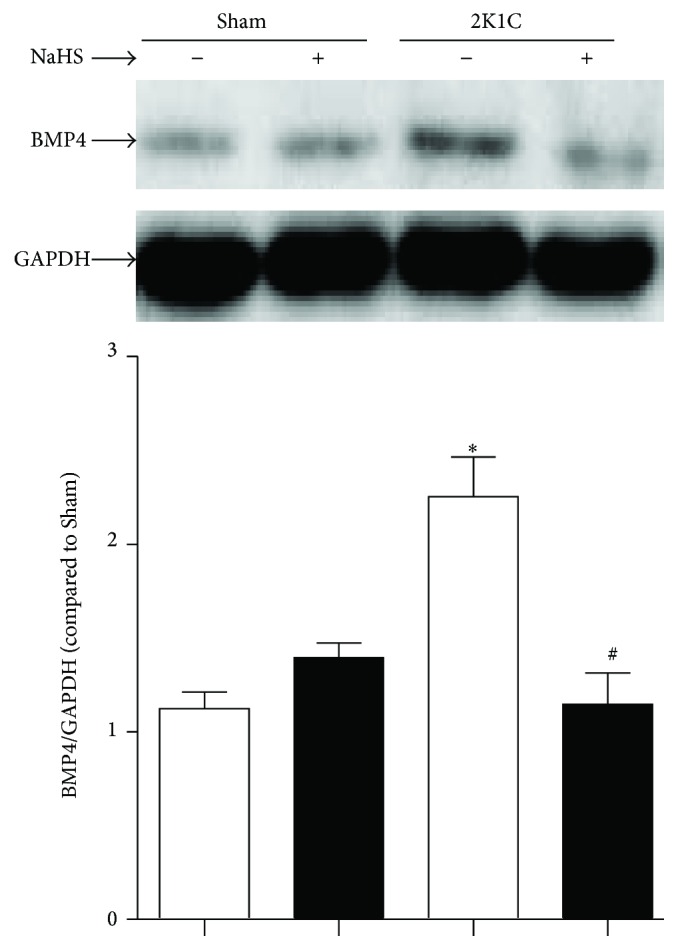
Effect of NaHS on protein levels of BMP4 in renal vascular hypertension rats. Data are means ± SEM. ^*∗*^ *P* < 0.05 versus Sham; ^#^ *P* < 0.05 versus 2K1C vehicle. *n* = 5 in each group.

The Western blot results showed that the protein expression of BMP4 was elevated in hypertensive renal artery. Chronic treatment with NaHS decreased the level of BMP4 in hypertensive rats (Figure 3).

### 3.4. Exogenous Administration of NaHS Decreased the Level of Oxidative Stress in Hypertensive Rats

**Figure 4:**
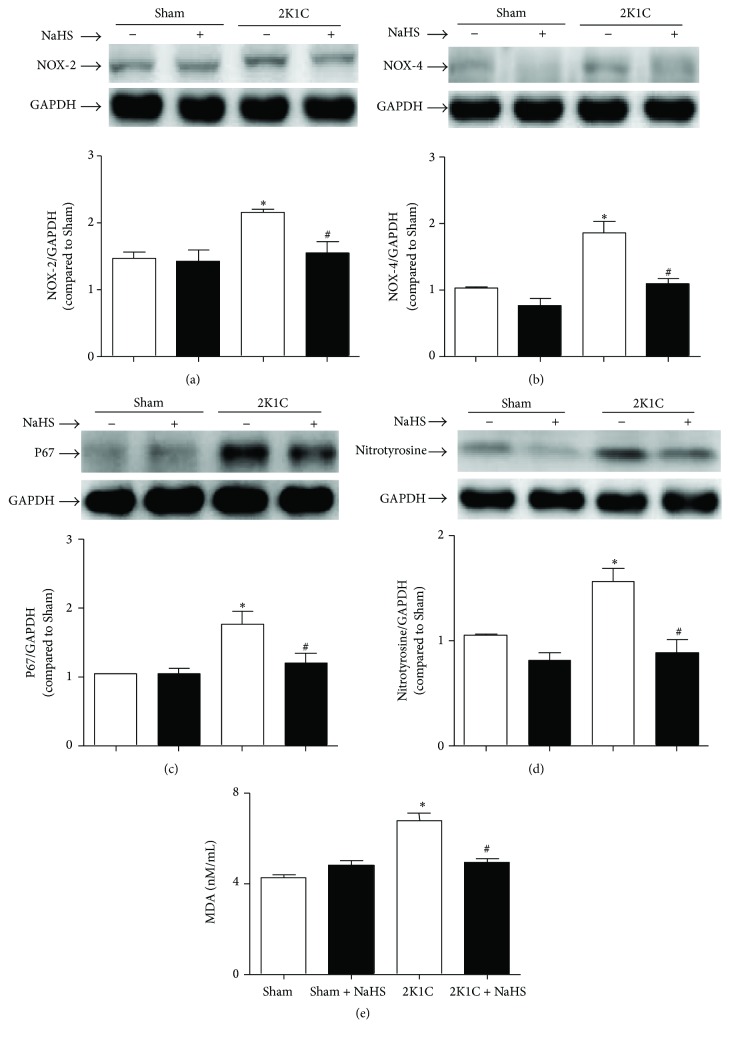
Effect of NaHS on oxidative stress. The oxidative stress was determined by the protein expression of NOX-2 (a), NOX4 (b), P67phox (c), and nitrotyrosine (d) in renal artery and the plasma level of MDA (e). Data are means ± SEM. ^*∗*^ *P* < 0.05 versus Sham; ^#^ *P* < 0.05 versus 2K1C vehicle. *n* = 5 in each group for Western blotting; *n* = 20 for MDA measurement.

The level of oxidative stress as reflected by the expressions of NOX-2, NOX-4, and p67^phox^ was augmented in 2K1C renal arteries. Exogenous treatment with NaHS rectified the overexpression of NOX-2, NOX-4, and p67^phox^ (Figures 4(a)–4(c)). As stable markers of oxidative stress, the level of nitrotyrosine in 2K1C arteries was increased and the plasma MDA level in 2K1C rats was significantly elevated (6.779 ± 0.3518 versus 4.273 ± 0.1313 nmol/mL, *P* < 0.05). Treatment with NaHS decreased the arterial protein levels of nitrotyrosine and the plasma level of MDA (4.947 ± 0.1649 versus 6.779 ± 0.3518 nmol/mL, *P* < 0.05) (Figures 4(d)-4(e)).

### 3.5. Exogenous Administration of NaHS Decreased Phosphorylation-p38 MAPK Protein Expression in Hypertensive Rats

**Figure 5:**
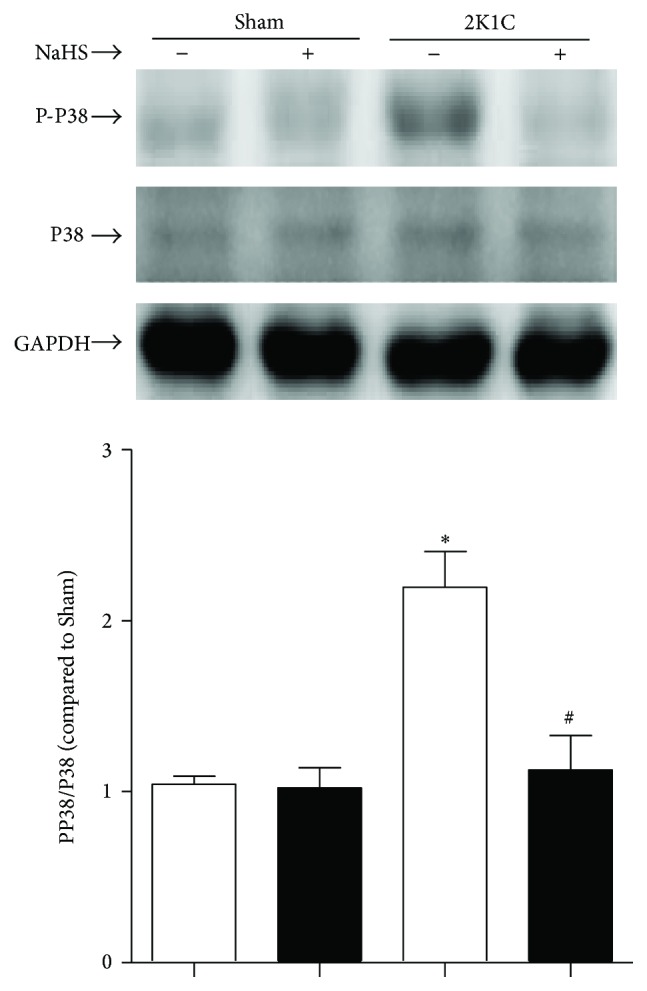
Effect of NaHS on the activation of p38 MAPK. Data are means ± SEM. ^*∗*^ *P* < 0.05 versus Sham; ^#^ *P* < 0.05 versus 2K1C vehicle. *n* = 5 in each group.

The Western blot results showed that the protein expression of the phosphorylation-p38 MAPK was upregulated in 2K1C renal arteries. Chronic treatment with NaHS could alleviate the increasing tendency of phosphorylation-p38 MAPK. However, there is difference on the total expression of p38 MAPK among all groups (Figure 5).

### 3.6. Exogenous Administration of NaHS Decreased COX-2 Protein Expression in Hypertensive Rats

**Figure 6:**
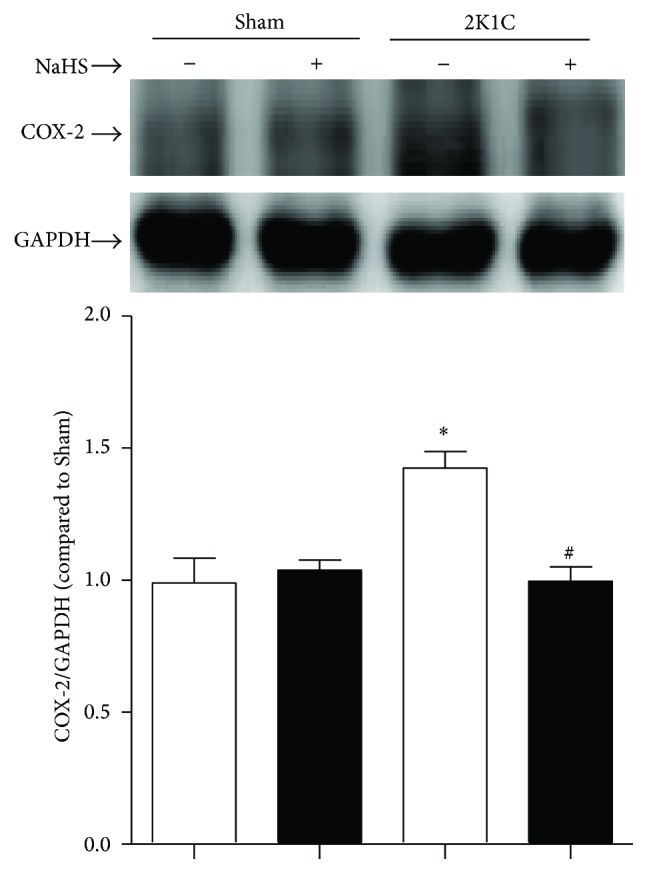
Effect of NaHS on protein levels of COX-2. Data are means ± SEM. ^*∗*^ *P* < 0.05 versus Sham; ^#^ *P* < 0.05 versus 2K1C vehicle. *n* = 5 in each group.

In renal arteries of 2K1C rats, protein expressions of COX-2 were increased compared with that of Sham rats. Chronic administration of NaHS inhibited the increased expression of COX-2 in 2K1C renal arteries (Figure 6).

## 4. Discussion

**Figure 7:**
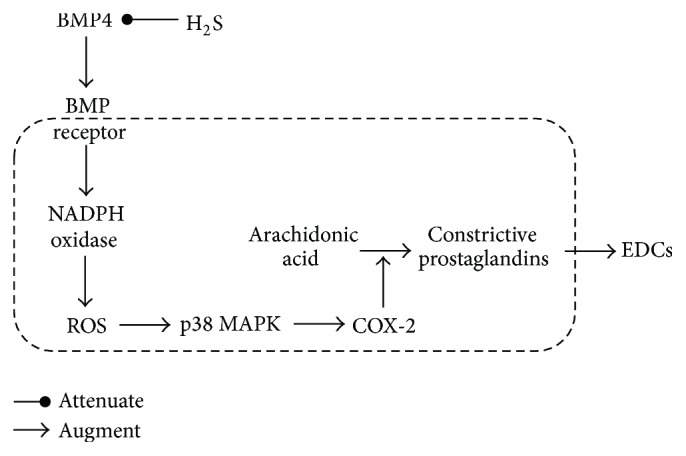
Schematic representation of the ameliorative effect of H_2_S on EDCs.

The present study demonstrates that NaHS reduces MAP and ameliorates EDCs which were both elevated in 2K1C hypertensive rats. Chronically administrating NaHS plays positive role in upregulating CSE protein expression and increasing the level of H_2_S in plasma, in which both of them were also reduced in 2K1C hypertensive rats. Moreover, we found that in hypertensive rats the protein levels of BMP4 are increased, and then oxidative stress and p38 MAPK are activated, resulting in upregulation of COX-2, where the pathway contributes to augmentation of EDCs. Intraperitoneal injection of NaHS interestingly reverses the activation of the above pathway (Figure 7).

Among variety of experimental or genetic models of hypertension, the 2K1C hypertensive model is a classical one of renovascular angiotensin-II-dependent hypertension [29]. The 2K1C rats represent transient activation of renin-angiotensin system (RAS) and thereafter sustained rise in blood pressure [30]. This model is used widely to investigate hypertension. Previous study shows that endogenous cystathionine-*γ*-lyase (CSE)/H_2_S pathway exited in vessels [31]. In hypoxia-induced pulmonary hypertension and maternal hypertension, endogenous CSE/H_2_S pathway was downregulated [32, 33]. In our experiment, we also find that 2K1C rats exhibit increasing MAP, decreasing plasma level of H_2_S, and downregulated protein expression of CSE. These results suggest that the hypertensive model has been established successfully, and endogenous CSE/H_2_S system is downregulated in hypertensive artery. Moreover, NaHS supplement lowers MAP and restores the impairment of CSE/H_2_S system. Our results demonstrate that endogenous CSE/H_2_S system serves as a critical factor in the pathogenesis of hypertension, which is in accordance with the results of published article [4].

Previous studies have demonstrated that impairment of endothelial function is tightly associated with pathogenesis of hypertension [34, 35]. The damaged endothelium manifested not only the deterioration of endothelium-dependent relaxation, but also the enhancement of the EDCs [24]. Our results show that EDCs was strengthened in renal arteries of 2K1C rats, whereas exogenous administration of NaHS reverses the enhanced EDCs in hypertension. These results suggest that H_2_S may ameliorate endothelial dysfunction and then reduces the elevated blood pressure.

Endothelial dysfunction induced by upregulation of NADPH oxidase and associated increasing oxidative stress has been found in spontaneously hypertensive rats [36, 37]. In experimental models of renovascular hypertension, the increased production of reactive oxygen species (ROS) mediates endothelial dysfunction resulting in progression of renovascular hypertension [38]. In an in vitro oscillatory shear stress (OS) model, it has firstly been confirmed that BMP4 coupled with oxidative stress [39]. A recent study also indicates that BMP4 can increase expression of NADPH oxidase and the level of ROS. These results suggest that BMP4 is a mediator and novel therapeutic target for cardiovascular diseases [40].

The endothelial dysfunction is due to the imbalance of the endothelium-derived relaxing and contracting factors [41, 42]. COX-2 is an inducible enzyme by inflammatory insult, and then its oxidative conversion of arachidonic acid in ECs results in the formation of an array of prostanoids that contributes to the occurrence of endothelium-dependent contractions [43, 44]. In renal arteries from hypertensive patients and SHR, BMP4 activates NADPH oxidase, leads to ROS overproduction and upregulation of COX-2 via p38 MAPK-dependent mechanism, which at last increases production of PGF2a, and then strengthens EDCs [24]. All of the above experiments indicated that BMP4/ROS/p38 MAPK/COX-2 pathway was involved in endothelium dysfunction of hypertension.

In our experiment, we find that the protein expressions of BMP4 are increased in hypertensive renal artery. The oxidative stress is accordingly strengthened in hypertension, verified by the increased expression of NADPH oxidase subtype, including NOX2, NOX4, and p67^phox^. Nitrotyrosine is considered as a biomarker for endogenous level of peroxynitrite [45] and has been correlated with elevated levels of other indices of oxidative stress [46]. Malondialdehyde (MDA) is formed in the lipid peroxidation caused by ROS and is also used as a biomarker to measure the level of oxidative stress. As stable marker of oxidative/nitrative stress [47], the expression of nitrotyrosine and the level of MDA elevated in 2K1C rats. Otherwise, the phosphorylation level of p38 MAPK is also increased in hypertensive renal artery. At last, protein expression of COX-2 is elevated in hypertensive renal artery. These results demonstrate that the activation of BMP4/ROS/p38 MAPK/COX-2 pathway involves the pathogenesis of EDCs and hypertension, in accordance with that in other published articles. Moreover, exogenous treatment of NaHS interestingly prevents the activation of the above pathways. These results suggest that protective effect of H_2_S on endothelium may be mediated by BMP4/ROS/p38 MAPK/COX-2 pathway.

Of course, there are several limits in our study. In our experiment, hypertension was induced by 2K1C animal model as previously described [48]. This model was used to mimic hypertension which is characterized by renin-angiotensin system (RAS) being excessively activated [49]. The results derived from the present study should be verified in other hypertensive animal models, such as spontaneous hypertension rats. Moreover, we only found the association between H_2_S and the BMP4/ROS/p38 MAPK/COX-2 pathway. The exact mechanism of how H_2_S regulates the above pathways needs to be further investigated.

## 5. Conclusion

Taken together, our present results demonstrate the inhibitory effect of H_2_S on BMP4 mediated cellular signaling cascade in hypertension, which may be involved in the ameliorative effect of H_2_S on endothelial dysfunction. Our findings further suggest the potential therapeutic value of H_2_S for hypertension.
